# Whole-genome sequencing reveals novel insights into sulfur oxidation in the extremophile *Acidithiobacillus thiooxidans*

**DOI:** 10.1186/1471-2180-14-179

**Published:** 2014-07-04

**Authors:** Huaqun Yin, Xian Zhang, Xiaoqi Li, Zhili He, Yili Liang, Xue Guo, Qi Hu, Yunhua Xiao, Jing Cong, Liyuan Ma, Jiaojiao Niu, Xueduan Liu

**Affiliations:** 1 School of Minerals Processing and Bioengineering, Central South University, Changsha, China; 2 Key Laboratory of Biometallurgy of Ministry of Education, Central South University, Changsha, China; 3 Institute for Environmental Genomics, University of Oklahoma, Norman, OK, USA

**Keywords:** *Acidithiobacillus thiooxidans*, Whole genome sequence, Bioinformatics analysis, Real-time quantitative PCR, Sulfur oxidation model

## Abstract

**Background:**

*Acidithiobacillus thiooxidans* (*A. thiooxidans*), a chemolithoautotrophic extremophile, is widely used in the industrial recovery of copper (bioleaching or biomining). The organism grows and survives by autotrophically utilizing energy derived from the oxidation of elemental sulfur and reduced inorganic sulfur compounds (RISCs). However, the lack of genetic manipulation systems has restricted our exploration of its physiology. With the development of high-throughput sequencing technology, the whole genome sequence analysis of *A. thiooxidans* has allowed preliminary models to be built for genes/enzymes involved in key energy pathways like sulfur oxidation.

**Results:**

The genome of *A. thiooxidans* A01 was sequenced and annotated. It contains key sulfur oxidation enzymes involved in the oxidation of elemental sulfur and RISCs, such as sulfur dioxygenase (SDO), sulfide quinone reductase (SQR), thiosulfate:quinone oxidoreductase (TQO), tetrathionate hydrolase (TetH), sulfur oxidizing protein (Sox) system and their associated electron transport components. Also, the sulfur oxygenase reductase (SOR) gene was detected in the draft genome sequence of *A. thiooxidans* A01, and multiple sequence alignment was performed to explore the function of groups of related protein sequences. In addition, another putative pathway was found in the cytoplasm of *A. thiooxidans*, which catalyzes sulfite to sulfate as the final product by phosphoadenosine phosphosulfate (PAPS) reductase and adenylylsulfate (APS) kinase. This differs from its closest relative *Acidithiobacillus caldus*, which is performed by sulfate adenylyltransferase (SAT). Furthermore, real-time quantitative PCR analysis showed that most of sulfur oxidation genes were more strongly expressed in the S^0^ medium than that in the Na_2_S_2_O_3_ medium at the mid-log phase.

**Conclusion:**

Sulfur oxidation model of *A. thiooxidans* A01 has been constructed based on previous studies from other sulfur oxidizing strains and its genome sequence analyses, providing insights into our understanding of its physiology and further analysis of potential functions of key sulfur oxidation genes.

## Background

*Acidithiobacillus thiooxidans* (*A. thiooxidans*), an extremely acidophilic, chemolithoautotrophic, gram-negative, rod-shaped microorganism, which are typically related to copper mining operations (bioleaching), has been well studied for industry applications. *A. thiooxidans* grows and survives by autotrophically utilizing elemental sulfur and reduced inorganic sulfur compounds (RISCs) as energy source [1], but it cannot use energy or electrons acquired from the oxidation of ferrous iron (Fe (II)) for carbon dioxide fixation as well as other anabolic processes [2].

Previous studies showed that the oxidation of elemental sulfur and RISCs was found in various strains of *Acidithiobacillus ferrooxidans* (*A. ferrooxidans*) through the detection of several enzymatic activities [3,4], but some of these activities were not associated with specific genes. The studies of oxidation and electron transfer pathways for elemental sulfur or RISCs are more complicated than those for ferrous iron, making the gene prediction and pathway clarification much more difficult [5]. Furthermore, some steps independent of enzymatic catalysis took place spontaneously, which adds the difficulty to modeling the mechanism of such a pathway. Fortunately, the method based on genome sequence analysis could provide the opportunities to predict some of these missing assignments, and also to suggest novel genes involved in the oxidation of elemental sulfur or RISCs such as sulfide, thiosulfate, and tetrathionate [6].

Elemental sulfur exists in the form of a stable octasulfane ring (S_8_) in nature, which forms orthorhombic crystals with extremely poor water solubility [7]. An activation prior to oxidation was postulated [6,8]. The first and critical step of sulfur oxidation could be an opening of the S_8_ ring by the thiol groups of cysteine residues, resulting in the formation of thiol-bound sulfane sulfur atoms (R-S-S_n_H) [8,9]. Subsequently, the R-S-S_n_H is transported into the periplasm and then oxidized by sulfur dioxygenase (SDO), of which gene(s) has (have) not yet been detected [6,9,10].

As sulfide was found to be one of the most common forms of S in the inorganic sulfur compounds, the initial step in RISCs oxidation mainly was the transition of sulfide into S^0^[11], forming a conjugated sulfur compound bound to a membrane fraction [5]. Furthermore, the first step of hydrogen sulfide oxidation is its conversion to sulfur or polysulfide in many phototrophic and chemotrophic bacteria by flavocytochrome c (Fcc), or by sulfide quinone reductase (SQR), which are located in the periplasm and the periplasmic surface of the cytoplasmic membrane, respectively [11,12]. Moreover, Fcc does not exist in various sulfide-oxidizing bacteria and appears to be restricted to certain species which possess the ability of thiosulfate oxidation [13].

Compared with the enzymes described above, the sulfur oxidizing (Sox) system has been elaborated in facultatively lithoautotrophic *Paracoccus pantotrophus* (*P. pantotrophus*). It is located in the periplasm and comprised of SoxXA (both c type cytochromes), SoxYZ (covalently sulfur-binding protein and sulfur compound chelating protein, respectively), SoxB (monomeric, dimanganese-containing protein that is similar to zinc-containing 5′-nucleotidases and considered to act as the sulfate thiol esterase component of the Sox system), and Sox (CD)_2_ (sulfur dehydrogenase), which mediate hydrogen sulfide-, sulfur-, thiosulfate- and sulfite-dependent cytochrome c reduction [14-17]. In contrast to the *P. pantotrophus*, the Sox (CD)_2_ complex is absent in the truncated Sox system of many α-Proteobacteria [17].

The periplasmic thiosulfate is synthesized spontaneously from sulfite and a sulfur atom [9], and then catalyzed to generate tetrathionate by thiosulfate:quinone oxidoreductase (TQO), which is constituted of a large subunit (DoxD) and a smaller subunit (DoxA); tetrathionate is then hydrolyzed by tetrathionate hydrolase (TetH) to produce thiosulfate and other uncertain products. TetH was studied previously in *A. ferrooxidans*[18], and the *tetH* gene cluster has been also characterized in *Acidithiobacillus caldus* (*A. caldus*) [19].

In addition, other RISCs oxidation enzymes were identified in *Acidithiobacillus* spp. including: rhodanese or thiosulfate sulfurtransferase (TST) and heterodisulfide reductase (HDR). The TST widely exists in the cytoplasm of both prokaryotes and eukaryotes. It cleaves the sulfur-sulfur bond of thiosulfate to yield sulfur and sulfite, and then the former is transferred to a thiophilic acceptor such as cyanide and thiol compounds [20,21]. The cytoplasmic heterodisulfide reductase complex HdrABC was reported to catalyze the reversible reaction of the disulfide bond X-S-S-X reduction accompanied with energy conservation in sulfate reducing archaea and bacteria and methanogenic archaea [2], while this complex was only speculated from transcriptomics and genomics analysis instead of biochemical experiments in *A. ferrooxidans*[9].

Recently, a model of electron transfer pathways involved in the sulfur oxidation of *A. ferrooxidans* was proposed, in which electrons released from RISC oxidation were transferred either to terminal oxidases to produce proton gradient or to NADH complex I to generate reducing power via the quinol pool (QH_2_) [2,6]. However, the lack of genetic manipulation systems has greatly restricted the exploration of the molecular biology and physiology in extreme acidophilic microorganisms, and considerably less information is known about the mechanism by which microorganisms grow, survive and proliferate in extremely acidophilic environments. With the ongoing and rapid development of sequencing technologies and the continuous improvement of bioinformatics-based analytical methods, effective tools have been offered for investigating metabolic and regulatory models [22]. A substantial body of information could be acquired by deep genome analysis, which can assist the laboratory scientists to focus on experimental investigation of several most significant predictions, thus save considerable time and efforts [23].

To get a better understanding of how these metabolic processes occur and further explore how to make them more efficient in *A. thiooxidans*, the whole-genome sequencing was carried out. Through bioinformatics analysis of the bioleaching bacterium, *A. thiooxidans* genome sequence, it is expected that we would predict and validate the genes and conserved gene clusters involved in sulfur oxidation. Subsequently, a further experiment at the transcriptional level was performed via quantitative real-time PCR (qRT-PCR). On the basis of bioinformatics analysis, together with qRT-PCR data, a putative model of sulfur oxidation in *A. thiooxidans* was proposed.

## Methods

### Ethics statement

The strain (*A. thiooxidans* A01) was obtained from a wastewater of coal dump of Jiangxi, China. This study doesn’t involve any ethical issue.

### Bioinformatics analysis of *A. thiooxidans* genome sequence

A bioinformatics pipeline was used to analyze the genome sequence of *A. thiooxidans*. The genomic DNA of *A. thiooxidans* A01 was extracted using TIANamp Bacteria DNA Kit (TIANGEN) according to the manufacturer’s instructions and then sequenced by BGI- Shenzhen (Beijing Genomics Institute) using Illumina HiSeq 2000 for 2 × 100 bp paired-end sequencing (Illumina, Inc. USA). After filtering, with Phred 20 as a cutoff, high quality raw sequences were assembled into longer fragment sequences, contigs and scaffolds, relied on strategy using SOAP*denovo* version 2.0 [24]. According to previous data, coding regions detection and potential genes identification were performed using Glimmer [25]. Moreover, RepeatMasker [26] was used to screen DNA sequences with interspersed repeats and low complexity. The RNAmmer [27] and tRNAscan-SE [28] were used to search for rRNA genes and tRNA genes in genomic sequence, respectively.

To further analyze the candidate genes and their predicted protein products, perl scripts written in our laboratory were used to extract the corresponding sequences of previously predicted CDSs. Subsequently, each putative gene was annotated using the BLASTx program (e value, ≤1e-5) against the alternative database such as Non-redundant protein database, Kyoto Encyclopedia of Genes and Genomes (KEGG), and Clusters of Orthologous Groups of proteins (COG).

### Media and culture conditions

The strain *A. thiooxidans* A01 was isolated in this laboratory. The components of 9 K medium [29] and DSMZ medium 71 [30] corresponding to S^0^ and Na_2_S_2_O_3_ media respectively were previously described in references. Elemental sulfur (S^0^) (boiling sterilized, 10 g/L) and Na_2_S_2_O_3_ · 5H_2_O (sterile filtration, 5 g/L) were added as substrates prior to inoculation. The bacterium was cultivated in 100-ml culture medium with an initial pH 2.0 for 9 K medium and pH 4.4 for DSMZ medium 71. The initial bacterial concentration was 2.5 × 10^6^ cells/ml and the cultivation temperature was 30°C. The shaking speed for liquid cultivation of *A. thiooxidans* A01 was 170 rpm if not specifically stated. All cultures under the same conditions were manipulated in triplicate.

### Quantitative real-time PCR (qRT-PCR)

Primers targeting selected genes putatively involved in sulfur oxidation were designed for quantitative real-time RT-PCR (product size 114–270 bp; Table 1). Cells were collected by centrifugation from 100 ml medium at the exponential growth phase (54 h) and washed twice using sterile RNase-free ddH_2_O. RNA was extracted using the Trizol Reagent method [31]. Total RNA extract was purified using MicroElute RNA Clean-Up Kit (OMEGA) in accordance with the manufacture’s recommendations, and the digestion of contaminating DNA was performed with RNase-free DNase I (OMEGA) to remove genomic DNA. RNA concentration and purity was measured at OD_260_ and OD_280_ with a NanoDrop ND-1000 spectrophotometer (NanoDrop Technologies).

**Table 1:** Primers used in qRT-PCR detection of genes related to sulfur oxidation

**Primer**		**Sequence (5′ to 3′)**	**Product size/bp**	**Anneal temp./°C**
**Name**	**Orientation**			
*sqr*	Forward	GCTCGGCAGCCTCAATAC	136	56
	Reverse	GGTCGGACGGTGGTTACTG		
*sor*	Forward	AAGCCCGTGCCTAAAGTG	266	56
	Reverse	CTGCCATAGTTGGTGTTGT		
*doxD*	Forward	CATCCCAGGACTCCACAA	223	56
	Reverse	GTCGCCACCTATTCTTACTATC		
*tetH*	Forward	TGAAAGACACGCTACCCG	270	56
	Reverse	GGCCGCTCAATGATAACC		
*hdrA*	Forward	CCGATTTGAAGGTGAAGC	185	56
	Reverse	CGGTTGCGACCATCTGTT		
*hdrB*	Forward	GTGGACCAGCGGGAAGAA	126	56.5
	Reverse	TACCACGGCTCTGGCATCG		
*hdrC*	Forward	TATTGAGTTTGGTCGCATTG	114	55.5
	Reverse	CCCTTGGACAGACGCTTT		
*soxA-I*	Forward	GCTCAGTCAGGGTAAGGC	161	56
	Reverse	GACAACTATTCAAACGCATC		
*soxB-I*	Forward	GCGTATTACCGATTTGCG	198	56
	Reverse	GGATTACCGGCCATGTTT		
*soxX-I*	Forward	GCAGGGTAATTGTTTGGC	163	56
	Reverse	CATATTGATGTGCGGGAT		
*soxY-I*	Forward	GGAATGTCAGCAGTGGGTAT	203	56
	Reverse	TTCTCCGCTATGGTTGGT		
*soxZ-I*	Forward	AAGCGGGCAAGTTGATTC	173	56
	Reverse	CGTATTGTCTTTCCAGGTC		
*soxA-II*	Forward	ATCTTGATGCCGTTGCTG	164	56
	Reverse	GCCCATTTCCCGACTTAT		
*soxB-II*	Forward	CCGTAAGGCATCACAGAG	244	56
	Reverse	CAAGGTATTAGCCCGTTT		
*soxX-II*	Forward	CACAAATAGTCGGCAACCT	237	56
	Reverse	CGCTCAGGGAAACTGTCTT		
*soxY-II*	Forward	TGATGCGTTGTTGGATGT	180	56
	Reverse	CGCCCACTATTGCTGAAAA		
*soxZ-II*	Forward	AGGTAGGGATTGGCACTG	120	56.5
	Reverse	CAAAGATAAGGCTGGAAAA		
*rhd*	Forward	GTGGTCCTGCTTACCCTCAA	130	56
	Reverse	GCCCGATAATATCCTGCTACTG		
*gapdh*	Forward	TAGCCCAGAACGCCTTTG	141	56
	Reverse	CGGTATGTCTTTCCGAGTG		

Subsequently, the total RNA of 2 μg was reversely transcribed using First Strand cDNA Synthesis Kit (TOYOBO) under the following conditions: 30°C for 10 min, 42°C for 20 min, 99°C for 5 min, and 4°C for 5 min. RT reaction products of 1 μl as template in 25 μl reaction volume were used for PCR amplification with specific primers (Table 1) using QuantiFast SYBR Green PCR Kit (QIAGEN). The conditions for the PCR reaction were as follows: 95°C for 5 min followed by 40 cycles at 95°C for 30 s, 56°C for 15 s and 72°C for 25 s in MyiQ Single-Color Real-Time PCR Detection System (BIO-RAD). The transcription reference gene, glyceraldehyde-3-phosphate dehydrogenase gene (*gapdh*), was used for normalization. The relative fold changes in gene expression were calculated using the 2^-ΔΔCT^ method [32].

### Nucleotide sequence accession numbers

The draft genome sequence of *A. thiooxidans* A01 was deposited at DDBJ/EMBL/GenBank under the accession number AZMO00000000. The version described in this paper is version AZMO01000000.

## Results and discussion

### Genomic properties

According to our sequencing data, the draft genome of *A. thiooxidans* A01 contains 3,820,158 total base pairs with GC content of 53.08% distributed in 213 contigs (Table 2). The maximum contig length is 259,764 bp, and the minimum length is 201 bp. The contig length has an N50 length of 46,830 bp. Compared to the draft genome of *A. thiooxidans* ATCC 19377 (AFOH00000000) in the NCBI database, the draft genome of *A. thiooxidans* A01 has much larger size, which indicates more information for gene prediction. In addition, 111 tRNA, one 5S-16S-23S operon and 3,660 protein-coding sequences (CDSs) were predicted. As to the 3,660 CDSs, 2,537 were assigned a putative function in the current databases, 136 were conserved hypothetical protein, and 987 were hypothetical proteins. Also, a total of 3,361 were involved in the KEGG pathways, and 2,664 were involved in the clusters of orthologous groups of proteins (COGs).

**Table 2:** **General features of draft genome sequence of *A. thiooxidans *** **A01**

**Characteristic**	**Value**
Total contigs	213
Total length (bp)	3,820,158
GC (%)	53.08
No. of tRNA genes	111
No. of rRNA operon (5S-16S-23S)	1
Total number of CDSs	3,660
Proteins with known function	2,537
Conserved hypothetical proteins	136
Hypothetical proteins	987

### Predicted genes involved in sulfur oxidation

Sulfur oxidation with sulfide, sulfur, sulfite, thiosulfate and tetrathionate as various oxidation states is the main pathway from a complete oxidation of sulfide to sulfate [33]. Based on genome sequence analysis, genes predicted to be involved in the oxidation of elemental sulfur and RISCs and electron transfer were detected in the genome (Table 3). It reveals that most of putative genes for sulfur oxidation [*sqr*, *sor,* two copies of *soxABXYZ, hdrABC, doxD, tetH* and rhodanese (*rhd*)] in *A. thiooxidans* also exist in *A. caldus*[11] and other microbial representatives derived from extreme acidic environments. Especially, the candidate genes potentially encoding sulfur dioxygenase (SDO) were detected in *A. thiooxidans* A01. In addition, *A. thiooxidans* also has genes encoding phosphoadenosine phosphosulfate (PAPS) reductase and adenylylsulfate (APS) kinase.

**Table 3:** **Amino-acid sequence identities of the products encoded by sulfur metabolic genes between *A. thiooxidans *** **A01 and other thiobacteria including *A. ferrooxidans*** **(CP001132),** ***A. caldus*** **(CP002573) and** ***T. denitrificans*** **(CP000116)**

**Gene**	**Position**	**Protein**	**aa identity (%)**		
			***A. ferrooxidans***	***A. caldus***	***T. denitrificans***
*orf1*	Contig14: 75514-76641	SQR	79	72	
*orf2*	Contig84: 6111-7199	TQO	62	76	
*orf3*	Contig84: 7212-8723	TetH	60	77	
*orf4*	Contig50: 1539-2474	SOR		80	
*orf5*	Contig102: 1875-2378	SoxY-I		76	
*orf6*	Contig102: 2425-2754	SoxZ-I		80	
*orf7*	Contig102: 2820-4529	SoxB-I		88	
*orf8*	Contig102: 7176-7559	SoxX-I		78	37
*orf9*	Contig102: 7588-8451	SoxA-I		79	36
*orf10*	Contig6: 24510-24905	SoxX-II		78	35
*orf11*	Contig6: 23962-24477	SoxY-II		76	37
*orf12*	Contig6: 23595-23927	SoxZ-II		84	40
*orf13*	Contig6: 22681-23544	SoxA-II		75	40
*orf14*	Contig6: 20337-22064	SoxB-II		86	51
*orf15*	Contig73: 12398-13453	HdrA	90	91	
*orf16*	Contig6: 1–918 (partial)	HdrB	95	95	
*orf17*	Contig6: 41616-42335	HdrC	95	90	
*orf18*	Contig19: 40873-41340	TST	74	74	
*orf19*	Contig175: 463–1311 (partial)	APS kinase	77	64	

**Figure 1:**
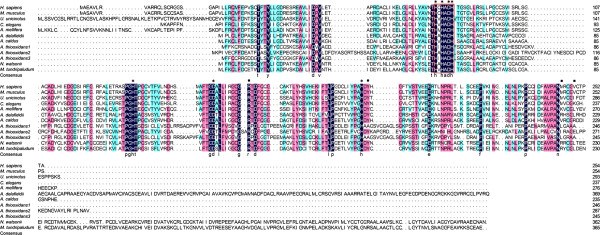
**Multiple sequence alignment among the SDO sequences from various species.** Conserved metal ion binding sites and potential GSH binding site are indicated by * and ■, respectively. The signature motif of metallo-beta-lactamase superfamily is marked with a red *rectangle.* GenBank accession number: *Homo sapiens* (NP_055112); *Mus musculus* (NP_075643); *Urechis unicinctus* (AEV92813); *Caenorhabditis elegans* (NP_501684); *Apis mellifera* (XP_393510); *Acidovorax delafieldii* (ZP_04761469); *Acidithiobacillus caldus* (YP_004749948); *Nitrosococcus watsonii* (YP_003760989); *Methylobacter tundripaludum* (ZP_08782165).

The first documented step in elemental sulfur oxidation is the transition of sulfur to thiosulfate, which is catalyzed by SDO. In *A. thiooxidans*, three putative *sdo* orthologs located at the draft genome sequence (contig8: 62452–63315, contig50: 14318–15058 and contig97: 6229–6966) belong to the large and considerably variable metallo-beta-lactamase superfamily (cl00446), which have the signature motif H-X-H-X-D-H (Figure 1). Previous research revealed that SDO in *Urechis unicinctus* (AEV92813) possessed the conserved metal I binding sites (H^113^, H^115^, H^169^ and D^188^), metal II binding sites (D^117^, H^118^, H^169^ and H^229^) and potential glutathione (GSH) binding sites (R^197^, Y^231^, M^279^ and I^283^) [34]. As is depicted in Figure 1, their conserved sites in At-SDO are much similar to AEV92813, and the possible reason why GSH binding sites between them is slightly different may be their distant relationship. Thus, the conserved regions observed with *U. unicinctus* as well as SDOs from other species possibly indicate the similar functional properties. However, the properties of SDO-like protein in *A. thiooxidans* are required further studies. Another gene encoding sulfide quinone reductase (SQR) was detected in the draft genome sequence of *A. thiooxidans* A01, and the product of *sqr* gene shares 79% and 72% identity with other *sqr* ortholog identified in *A. ferrooxidans* and *A. caldus*, respectively (Table 3).

**Figure 2:**
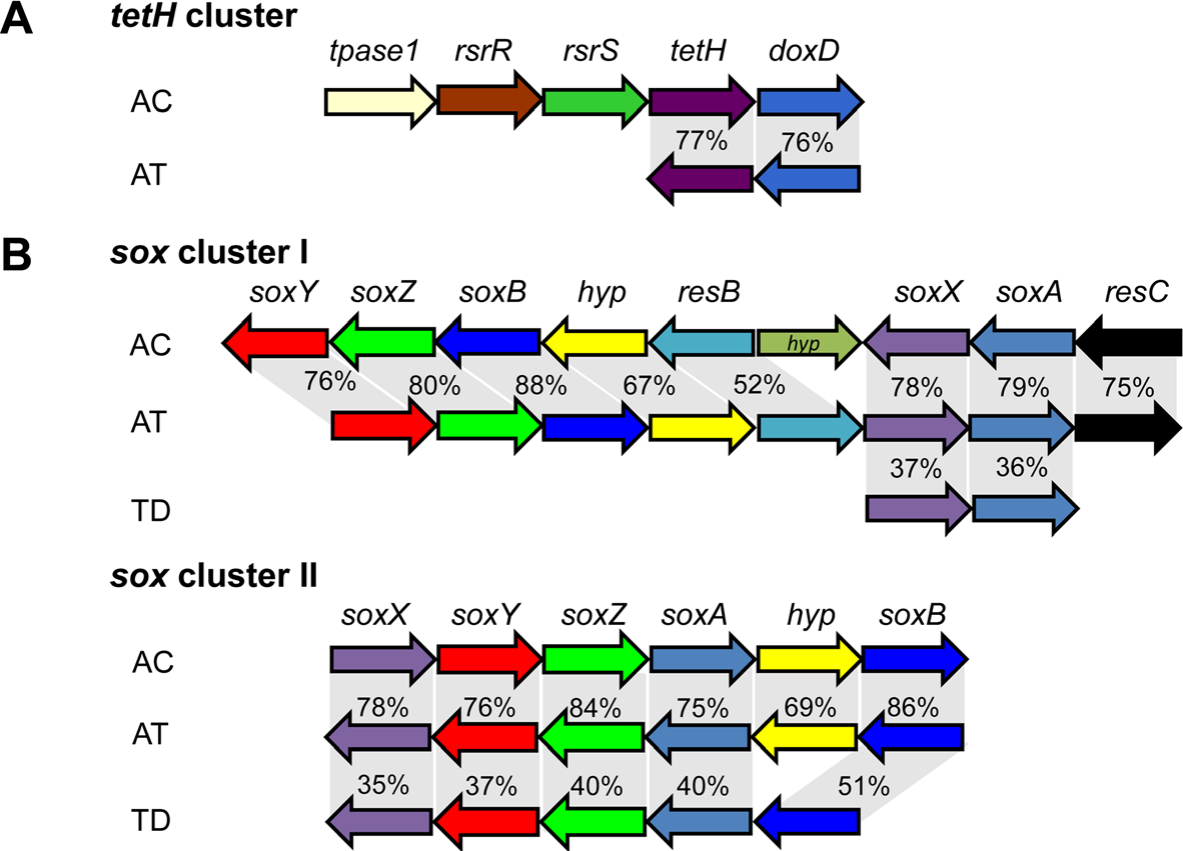
**Comparison of *tetH *gene cluster (A) and the *sox *gene clusters (B) between *A. thiooxidans *A01 and other sulfur oxidizers containing identified or putative genes.** Abbreviations: *tpase*, transposase; *rsrR*, response regulator (two-component system); *rsrS*: histidine kinase (two-component system); *tetH*, tetrathionate hydrolase; *doxD*, thiosulfate:quinone oxidoreductase; *hyp*, hypothetical protein; *resB*, cytochrome c-type biogenesis protein ResB; *resC*, cytochrome c-type biogenesis protein ResC. The *tetH* cluster in AT: *A. thiooxidans* A01, AC: *A. caldus* SM-1 (CP002573); Two sox clusters in AT: *A. thiooxidans* A01, AC: *A.caldus* SM-1 (CP002573), TD: *T. denitrificans* ATCC 25259 (CP000116). The homology proteins are expressed by the same color, and percentage of amino acid similarity is indicated. The direction of transcription is represented by the arrows.

Other enzymes reported to be involved in sulfur oxidation are TQO and TetH. Our sequence analysis revealed that the homolog of *doxDA* existed in the draft genome of *A. thiooxidans* A01, and it is predicted to encode a thiosulfate:quinone oxidoreductase (TQO), and also has a conserved DoxD domain (pfam04173) and a conserved DoxA domain (pfam07680). There is a fusion of separate DoxD- and DoxA–like subunits that were reported previously in *A. ferrooxidans* DoxD [6]. The putative TetH of *A. thiooxidans* shares 60% and 77% identity with TetH in *A. ferrooxidans* and *A. caldus* respectively, indicating their high similarity in orthologous relationship. Our analysis also indicates that TetH of all sequenced *Acidithiobacillus* spp. has a conserved pyrrolo-quinoline quinone (PQQ) domain (pfam01011). Although TetH was predicted to be external membrane proteins, experimental evidence showed that it was a soluble periplasmic homo-dimer with an optimum pH of 3 in *A. caldus*[35]. Previous studies have revealed that there is a *tetH* gene cluster in *A. caldus*[19], which is comprised of five cotranscribed genes, *tpase1*, *rsrR*, *rsrS*, *tetH* and *doxD*. While in the draft genome sequence of *A. thiooxidans* A01, only *tetH* and *doxD* located at the upstream constitute the *tetH* cluster (Figure 2A).

In addition, bioinformatics analysis identified a truncated Sox sulfur oxidizing system in *A. thiooxidans* A01, which contains two *sox* gene clusters, *sox* cluster I (*resC-soxAX-resB-hyp-soxBZY*) and *sox* cluster II (*soxXYZA-hyp-soxB*), and both are quite differ from those of *Paracoccus denitrificans*, *Pseudaminobacter salicylatoxidans* and *Starkeya novella*: *soxRSVWXYZABCDEFGH* (X79242), *soxGTRSVWXYZABCD* (AJ404005) and *soxFDCBZYAXWV* (AF139113) [14,15,36,37]. Comparison of all genes sequences in two *sox* clusters of *A. thiooxidans* A01 to those of *Thiobacillus denitrificans* ATCC 25259 (CP000116) revealed amino-acid sequence identities in the range of 35% to 51%. Unlike the *sox* cluster II of *A. thiooxidans* A01, however, there is only additional *soxXA* in the genome sequence of *T. denitrificans* ATCC 25259 [37]. Interestingly, significant sequence similarity (52% to 88%) and relatively conserved gene constitution of *A. thiooxidans* A01 *sox* gene clusters to those of *A. caldus* SM-1 (CP002573) indicated the similar functional properties (Figure 2B).

Furthermore, heterodisulfide reductase (HDR) has been postulated to be involved in sulfur oxidation. All genes (*hdrA*, *hdrB* and *hdrC*) of HDR were found in the draft genome of *A. thiooxidans* A01. The putative HdrA subunit is flavoprotein within a FAD binding domain and a NAD (P)-binding Rossmann-like domain. The HdrB subunit contains the typical cysteine-rich domain and the remaining HdrC subunit contains 4Fe-4S dicluster domain. In addition, all putative Hdr subunits in *A. thiooxidans* A01 suggested over 90% identities to the respective Hdr subunits compared with *A. ferrooxidans* and *A. caldus* (Table 3).

Five genes encoding rhodanese (sequence length 109 aa to 155 aa) were detected and one of them was used to design primer for qRT-PCR (Table 1). The phosphoadenosine phosphosulfate (PAPS) reductase and adenylylsulfate (APS) kinase, which consecutively oxidize sulfite to produce sulfate via an indirect pathway, were found in the genome of *A. thiooxidans.* Two genes encoding APS kinase and three genes encoding PAPS reductase were predicted in the draft genome but their roles in sulfur oxidation remain to be established.

The genome information showed that *A. thiooxidans* A01 contains two gene clusters potentially encoding components of the NADH quinone-oxidoreductase complex (*nuoABCDEFGHIJKLMN*) similar to that in *A. caldus*[11]*.* In addition, seven copies of *bd* ubiquinol oxidase genes (*cyd*AB) and two gene clusters encoding *bo*_*3*_ ubiquinol oxidase (*cyoB*ACD) exist in *A. thiooxidans* A01. However, components of the *aa*_*3*_-type cytochrome oxidase genes (*cox*BACD) only exist in the *A. ferrooxidans*[6].

### The sulfur oxygenase reductase gene (*sor*) found in *A. thiooxidans*

As the initial enzyme in the aerobic sulfur oxidation of thermophilic archaea [38,39] and acidophilic bacteria [40,41], the sulfur oxygenase reductase (SOR) has been identified. SOR simultaneously catalyzes elemental sulfur to produce sulfite, thiosulfate, and sulfide [38,42], which are oxygen-dependent disproportionation reactions. The catalytic reaction has certain characteristics: (1) The enzyme involved in this reaction is soluble and located in cytoplasm [43]; (2) The optimum pH and temperature for activity are founded to be 7.0 ± 0.5 (*Sulfolobus brierleyi*) and 65–85°C (*Acidianus tengchongensis* and *Acidianus ambivalens*), respectively [44,45]; and (3) It does not require cofactors or external electron donors/acceptors for activity [46,47].

**Figure 3:**
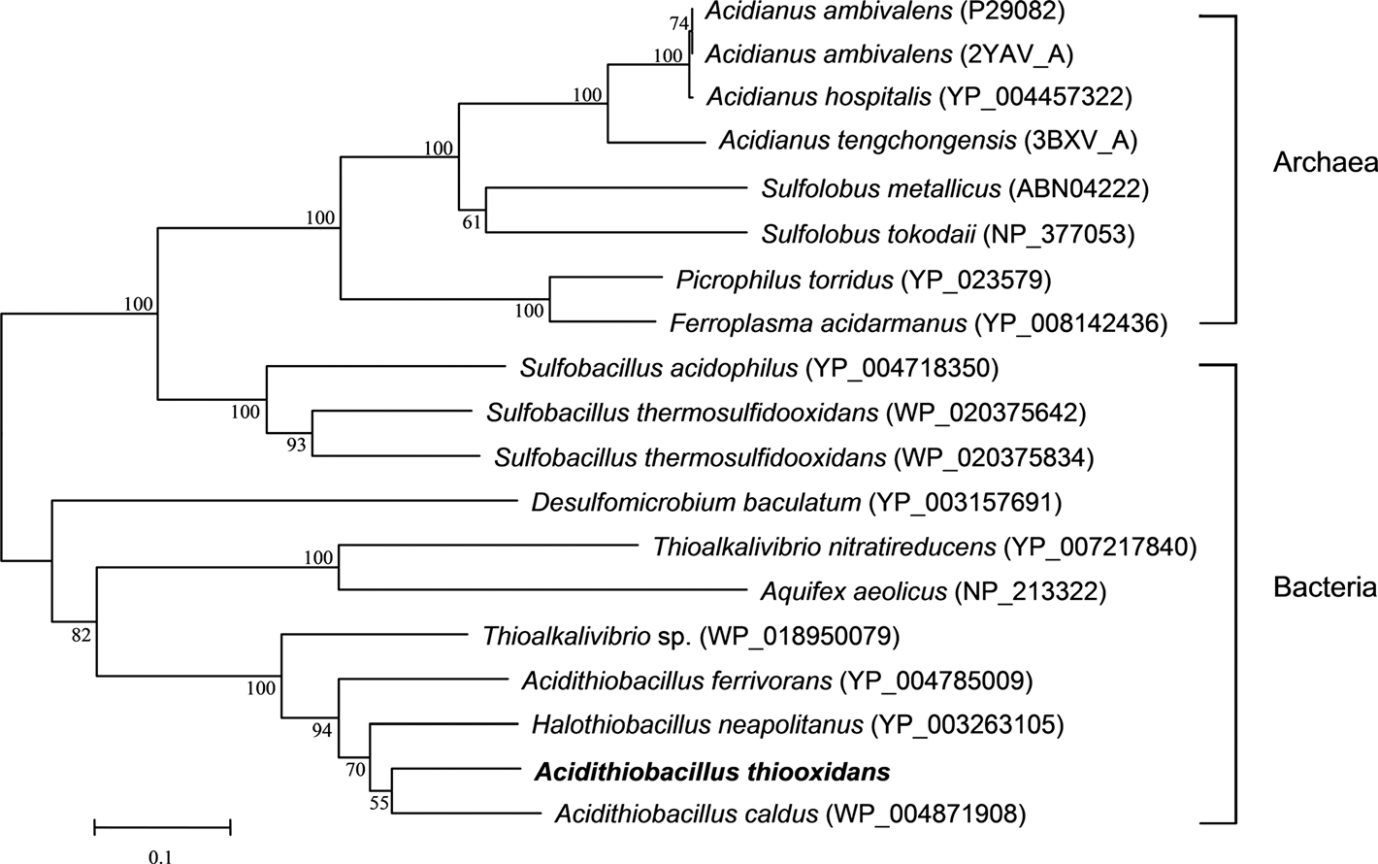
**Phylogenetic dendrogram based on the SORs from *A. thiooxidans *and its close homologs from other species.** It was constructed using the neighbor-joining algorithm implemented in the MEGA version 5.05, and its reliability was evaluated by 1,000 bootstrap replicates.

The sulfur oxidation system based on the sulfur oxygenase reductase was only reported in several acidophilic and thermophilic archaea (e.g., *A. ambivalens*, *A. tengchongensis*) or bacteria (e.g., *A. caldus*), but not in the species of *A. ferrooxidans* and *A. thiooxidans*[9,17,41,48]*.* However, our annotations show that a potential gene encoding SOR with very similar amino acid sequence in *A. caldus*, was detected in *A. thiooxidans* A01. Interestingly, the *sor* gene was not found in the draft genome sequence of *A. thiooxidans* ATCC 19377 [49]. To date, it is unclear whether *sor* gene exists or not in *A. thiooxidans* ATCC 19377 unless the complete genome sequence is obtained. Subsequently, homology search was performed with BLASTx, and sequence analysis indicated that the putative enzyme of *A. thiooxidans* A01 shared 80% identity to the SOR of *A. caldus*. Moreover, the phylogenetic tree, which included almost all homologs of SOR derived from BLASTp search showed that SOR from *A. thiooxidans* was detected to be closest to that isolated from *A. caldus*. High bootstrap values insure the reliability of clustering (Figure 3).

**Figure 4:**
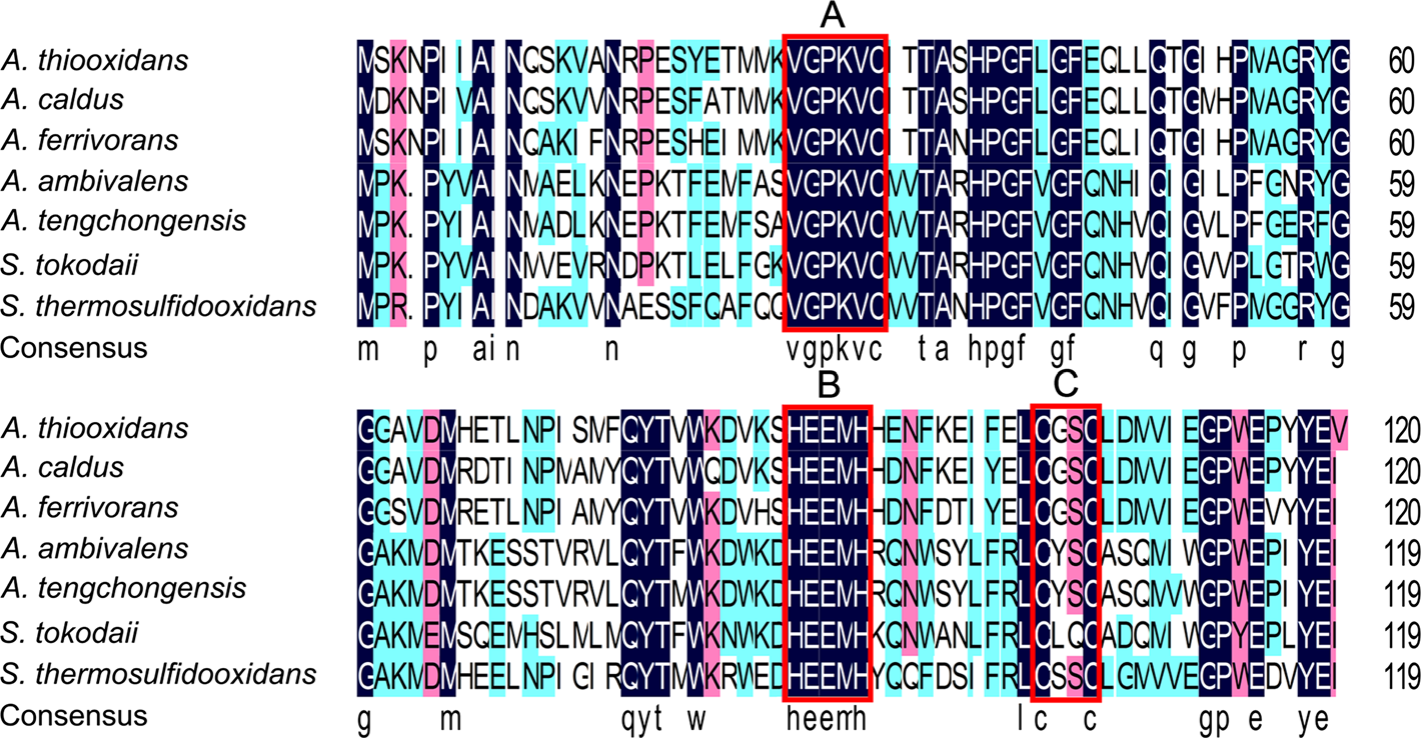
**Multiple sequence alignment of SORs between *A. thiooxidans *and other species.** Protein sequences were aligned by the DNAMAN multiple sequence alignment program. The putative domains in SOR were *boxed by the rectangle*. And the conserved domains V-G-P-K-V-C^32^, H^87^-E-E-M-H^91^ and C^102^-XX-C^105^ were indicated by the *rectangle***A**, **B**, **C**, respectively. Abbreviations: *A. thiooxidans, Acidithiobacillus thiooxidans; A. caldus, Acidithiobacillus caldus* (WP_004871908); *A. ferrivorans, Acidithiobacillus ferrivorans* (YP_004785009); *A. ambivalens, Acidianus ambivalens* (P29082)*; A. tengchongensis, Acidianus tengchongensis* (AAK58572); *S. tokodaii, Sulfolobus tokodaii* (NP_377053)*; S. thermosulfidooxidans*, *Sulfobacillus thermosulfidooxidans* (WP_020375642).

In addition, homology searches showed that the nucleotide sequence of this putative gene in *A. thiooxidans* and its predicted amino acid sequence have high similarities to other species. Multiple sequence alignment of SORs from *A. thiooxidans* and other species was carried out (Figure 4). It is demonstrated that three cysteine residues located in two separately conserved domains, C^32^ at V-G-P-K-V-C^32^ and C^102^ and C^105^ at C^102^-X-X-C^105^, are essential to its activity [50]. In addition, the conserved motif H^87^-X_3_-H^91^-X_23_-E^115^, which is considered to be iron binding site [47], is detected in SOR from *A. thiooxidans*. The crystal structure of the SOR in *A. ambivalens* is demonstrated to be a large homo-multimer composed of 24 identical monomers of 308 residues, forming a large hollow sphere [51,52]. The active sites of SOR are constituted of a mononuclear non-heme iron site and three conserved cysteine residues [45]. Furthermore, the structural analysis has been performed to study the potential functions of the cysteine residues in *A. tengchongensis*. It is proposed that C^32^ residue constitutes most possibly the substrate binding site and that C^102^ and C^105^, together with the iron binding motif H^87^-X_3_-H^91^-X_23_-E^115^, probably form the catalytic site.

So far as we know, the *sor* gene is for the first time detected and reported in *A. thiooxidans*. The discovery of *sor* gene supplies a novel idea that these genome-based predictions can offer new opportunities to detect similar genes in other microorganisms and also provide new markers to explore the metabolic pathways. However, the further biochemical experiments at the transcription and protein levels should be carried out in order to verify the presence or absence of *sor* gene.

### Growth of *A. thiooxidans* in S^0^ and Na_2_S_2_O_3_ media

**Figure 5:**
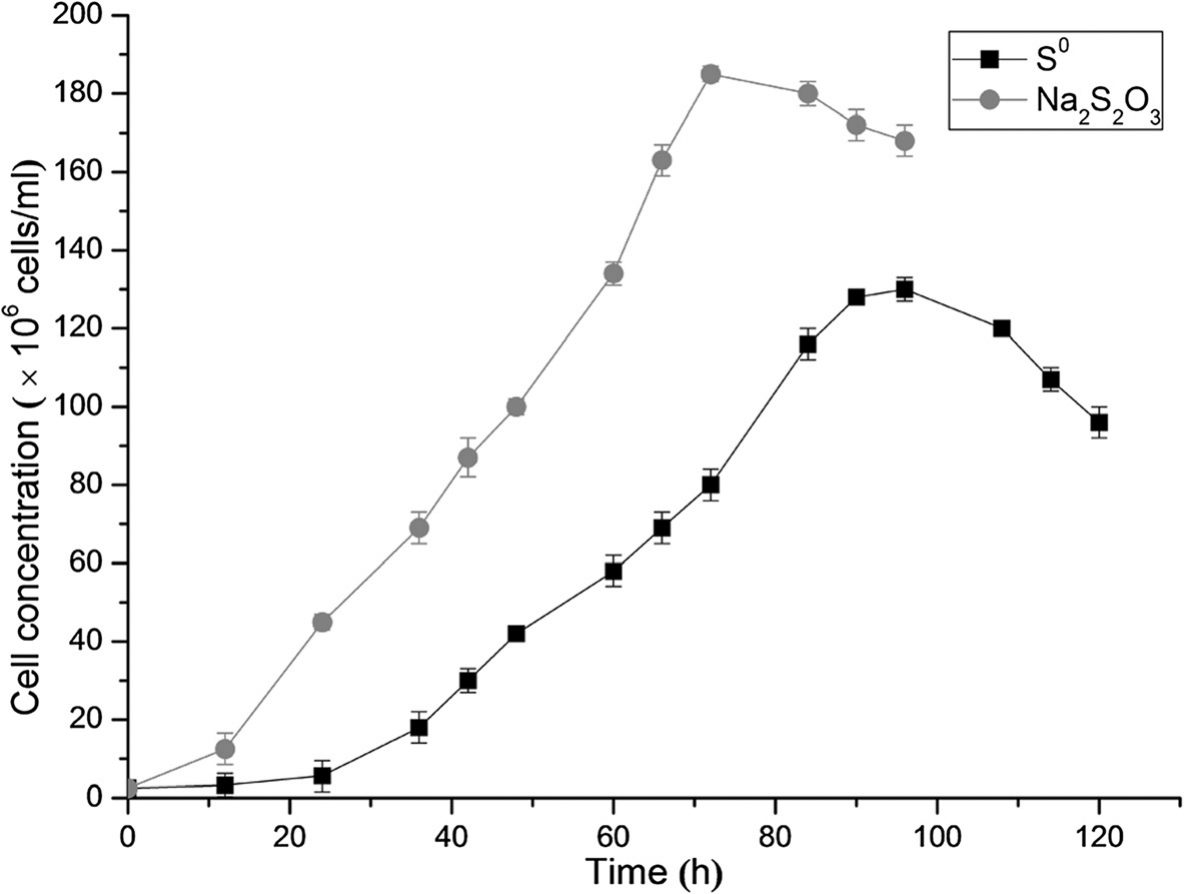
**The growth curve of *Acidithiobacillus thiooxidans *A01 in S**^**0 **^**medium and Na**_**2**_**S**_**2**_**O**_**3 **_**medium.** Data are average of three growth experiments.

To examine the growth of *A. thiooxidans* A01, Starkey-S^0^ or Starkey-Na_2_S_2_O_3_ was used as the substrate in liquid media. The results showed that *A. thiooxidans* had the ability to utilize both S^0^ and Na_2_S_2_O_3_ as the energy sources (Figure 5). Furthermore, the soluble Na_2_S_2_O_3_ was used prior to S^0^ and bacteria in the Na_2_S_2_O_3_ medium reached stationary phase earlier than that in the S^0^ medium. Moreover, the cell concentration of *A. thiooxidans* in the Na_2_S_2_O_3_ medium was obviously higher than that in the S^0^ medium, suggesting that *A. thiooxidans* has a highly efficient thiosulfate oxidizing ability to enable it to grow better with Na_2_S_2_O_3_ as substrate. One of possible reasons is that Na_2_S_2_O_3_ can more easily and quickly enter into the organism and then be used as energy source, while S^0^ needs to be activated before it is transferred into the periplasm, resulting in a slower growth and lower cell concentration with S^0^ as substrate.

### Expression of selected genes involved in sulfur oxidation

To understand how those identified key genes involved in sulfur oxidation are expressed with Na_2_S_2_O_3_ and S^0^ as substrates, we further examined the expression of the selected sulfur oxidation genes with Na_2_S_2_O_3_ as substrate compared to those with S^0^ as substrate using qRT-PCR (Table 4). The *gapdh* gene was used as the internal control to regulate the systematic and random errors during the process of operation procedure. Statistical significance was assessed by the Student’s *t*-test, and the gene expression levels with fold change ≥ 1.50 and p-value ≤ 0.05, or with fold change ≤ 0.67 and p-value ≤ 0.05 were up-regulated and down-regulated, respectively. Our results showed that all assayed genes were transcribed when grown in S^0^ or Na_2_S_2_O_3_, and *t*-test analysis revealed the significant (p < 0.05) differences in gene expression between S^0^- and Na_2_S_2_O_3_-grown cells were observed. Although no obvious changes at the transcription level were detected for *tetH*, *hdrA* and the *sox* gene cluster II (e.g., *soxA*-*II*, *soxB*-*II* and *soxY*-*II*), the other genes (e.g., *sqr*, *sor* and *tqo*) were down-regulated at various degrees with Na_2_S_2_O_3_ as substrate (Table 4). Based on the growth curve and qRT-PCR data, it is speculated that soluble Na_2_S_2_O_3_ could quickly enter into the cell and then stimulate the up-regulation of genes involved in sulfur oxidation at the early stage, whereas the accumulation of products derived from sulfur oxidation in turn might inhibit the process of enzyme reactions and then influence the gene expression. With S^0^ as substrate, however, elemental sulfur needs to be activated before through the outer membrane, and the majority of sulfur oxidation genes including *sor* gene, which was detected to be the low expression level at the early growth phase in *A. caldus*[9], would play an important role till the mid-log phase.

**Table 4:** **Comparison of raw fold changes in gene expression between S**^**0 **^**and Na**_**2**_**S**_**2**_**O**_**3**_**-grown cells (mid-log phase) obtained by qRT-PCR in this study**

**Gene**	**Function**	**2**^**-ΔΔCT **^**(S**^**0**^**/Na**_**2**_**S**_**2**_**O**_**3**_**)**
Sulfide-quinone reductase		
*sqr*	Sulfide quinone reductase	4.14 ± 0.09
sulfur oxygenase reductase		
*sor*	Sulfur oxygenase reductase	4.06 ± 0.01
tetrathionate hydrolase operon		
*doxD*	Thiosulfate:quinone oxidoreductase subunit	3.19 ± 0.01
*tetH*	Tetrathionate hydrolase	0.96 ± 0.06
Heterodisulfide reductase complex operon		
*hdrA*	Pyridine nucleotide-disulfide oxidoreductase	1.35 ± 0.02
*hdrB*	Heterodisulfide reductase subunit B	171.37 ± 0.09
*hdrC*	Iron-sulfur cluster-binding protein	49.69 ± 0.02
Sox operon I		
*soxA-I*	Diheme cytochrome c	48.17 ± 0.01
*soxB-I*	Sulfate thiol esterase	4.98 ± 0.11
*soxX-I*	Cytochrome c, class I	13.80 ± 0.08
*soxY-I*	Covalently sulfur-binding protein	3.89 ± 0.01
*soxZ-I*	Sulfur compound-chelating protein	150.12 ± 0.06
Sox operon II		
*soxA-II*	Diheme cytochrome c	1.23 ± 0.04
*soxB-II*	Sulfate thiol esterase	1.42 ± 0.08
*soxX-II*	Cytochrome c, class I	1.57 ± 0.07
*soxY-II*	Covalently sulfur-binding protein	0.83 ± 0.04
*soxZ-II*	Sulfur compound-chelating protein	1.87 ± 0.04
Rhodanese (sulfur transferase)		
*rhd*	Rhodanese	13.04 ± 0.08

Shown are the means of fold change from qRT-PCR of three to four replicates with standard deviations. Genes whose fold change ≥ 1.50 with p-value ≤ 0.05 and fold change ≤ 0.67 with p-value ≤ 0.05 are considered up-regulation and down-regulation, respectively.

With S^0^ in the medium, a mass of sulfur atoms (S) and hydrogen sulfide are produced during the activation of S_8_ and then are used as the substrates of SQR, SOR, TST and HDR in succession. This could be the reason for the higher expression of *sqr*, *sor*, *rhd* and *hdrABC* in the S^0^ medium than in the Na_2_S_2_O_3_ medium (Table 4). Our experiments showed that thiosulfate was sufficient at the mid-log phase in the Na_2_S_2_O_3_ medium, thus resulting in the lower expression level of *doxD* and *tetH*. The *sox* operon I but not *sox* operon II together with a *bo*_*3*_ ubiquinol oxidase operon gene may make it feasible to regulate and control at their transcriptional level, thus this may be one of the reasons why the expression of the *sox* operon I was up-regulated in the S^0^ medium whereas the sox operon II had no obvious changes in these two substrates.

### Construction of sulfur oxidation model in *A. thiooxidans*

**Figure 6:**
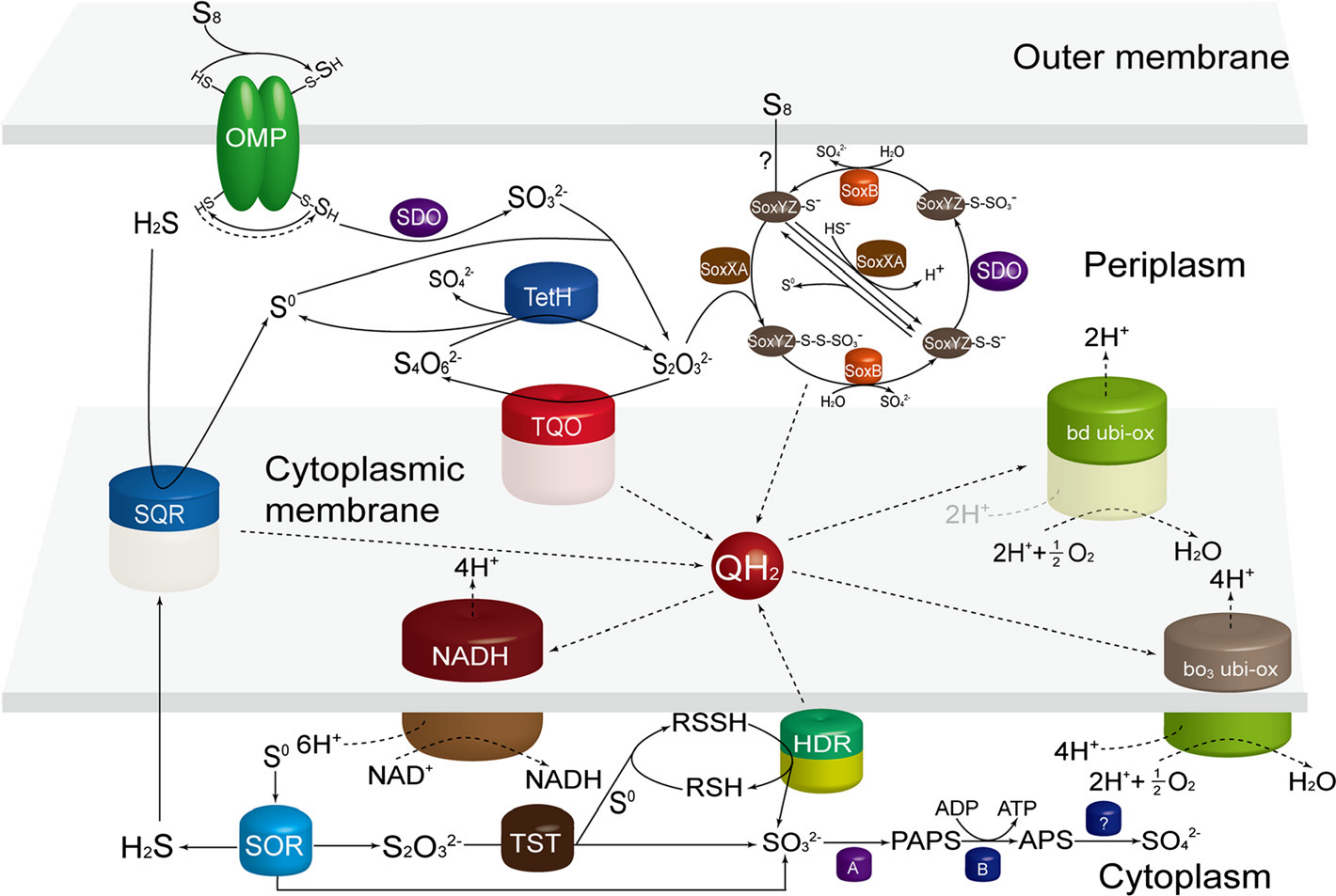
**The sulfur oxidation model in *A. thiooxidans *based on documented models [**[2]**,**[6]**,**[9]**-**[11]**], bioinformatics analysis of draft genome sequence.** Abbreviations: SDO, sulfur dioxygenase; SQR, sulfide quinone reductase; TQO, thiosulfate:quinone oxidoreductase; TetH, tetrathionate hydrolase; Sox, sulfur oxidizing protein; HDR, heterodisulfide reductase; SOR, sulfur oxygenase reductase; TST, thiosulfate sulfurtransferase; A, phosphoadenosine phosphosulfate reductase; B, adenylylsulfate kinase. As is shown, the sulfur oxidation metabolism in *A. thiooxidans* A01 contains various sulfur oxidation systems and the electron transfer pathways in different cellular compartments. (i) In the outer membrane, elemental sulfur (S_8_) in the form of stable octasulfane ring is activated and transported into the periplasm as thiol-bound sulfane sulfur atoms (R-S-S_n_H); (ii) In the periplasm, R-S-S_n_H is oxidized by SDO, while tetrathionate is utilized by TetH to produce thiosulfate, and the thiosulfate is sequentially catalyzed by Sox complex; (iii) In the cytoplasmic membrane, SQR and TQO carry out their respective functions with electrons further transferred to terminal oxidase or NADH complex I; and (iv) In the cytoplasm, the particular enzymes perform the catalytic reaction by a sequence of steps that eventually produce sulfate.

In order to acquire the functional attributes of cells, it is necessary to understand the structural constitution and characters of cellular metabolic networks [53]. With respect to sulfur oxidation, a bioinformatics analysis of the genome sequence of organism was performed, indicating that various enzymes, enzyme complexes, and the electron transport chain components were located in different cellular compartments. Based on the documented models in other *Acidithiobacillus* species [2,6,9-11], genome sequence analysis, our current knowledge, and experimental results in this study, a hypothetical model is developed for sulfur oxidation in *A. thiooxidans* A01 (Figure 6).

The first documented step in sulfur oxidation system is the activation of extracellular elemental sulfur (S_8_) to thiol-bound sulfane sulfur atoms (R-S-SH) and then it is transferred into the periplasm where it is oxidized by the sulfur dioxygenase (SDO) to produce sulfite (Table 5), accompanied with the generation of hydrogen sulfide. Subsequently, hydrogen sulfide could be converted to sulfur atoms (S) by sulfide quinone reductase (SQR) located in the cytoplasmic membrane. The sulfur atoms could be accumulated to form polymeric sulfur (S_n_), and then transferred via an unknown mechanism into the cytoplasm where it is catalyzed by sulfur oxygenase reductase (SOR). The possible products of disproportionation reaction performed by SOR are sulfide, thiosulfate and sulfite. Sulfide could be converted to hydrogen sulfide and then oxidized by SQR, while thiosulfate is considered to be used as the substrate of rhodanese (TST) to produce sulfite and sulfur atom. Subsequently, the cytoplasmic thiol protein (RSH) that is acted as sulfur atom acceptor obtains a sulfur atom to form sulfane sulfate (RSSH), and then the latter is catalyzed by the heterodisulfide reductase complex (HDR) to regenerate RSH. Therefore, a cycle relied on TST and HDR is proposed: RSH acquires a sulfur atom derived from the catalysis of thiosulfate, which is catalyzed by TST, to generate RSSH, and then RSSH is used as the substrate of HDR to reproduce RSH [9].

**Table 5:** **Enzyme properties involved in the sulfur oxidation of *A. thiooxidans***

**Abbreviation**	**Enzyme name**	**No. of enzyme**	**Position**	**Reaction**
SDO	Sulfur dioxygenase	EC 1.13.11.18	Periplasm	S^0^ → SO_3_^2−^
SQR	Sulfide quinone reductase	EC 1.8.5.4	Inner Membrane	H_2_S → S^0^
TQO	Thiosulfate:quinone oxidoreductase	EC 1.8.5.2	Inner Membrane	S_2_O_3_^2−^ → S_4_O_6_^2−^
TetH	Tetrathionate hydrolase		Periplasm	S_4_O_6_^2−^ → S_2_O_3_^2−^ + SO_4_^2−^ + S^0^
Sox	Sulfur oxidizing protein		Periplasm	S_2_O_3_^2−^ → SO_4_^2−^ + S^0^
HDR	Heterodisulfide reductase		Cytoplasm	RSSH → RSH + SO_3_^2−^
SOR	Sulfur oxygenase reductase	EC 1.13.11.55	Cytoplasm	S^0^ → H_2_S + SO_3_^2−^ + S_2_O_3_^2−^
TST	Thiosulfate sulfurtransferase	EC 2.8.1.1	Cytoplasm	S_2_O_3_^2−^ → SO_3_^2−^ + S^0^
PAPS reductase	Phosphoadenosine phosphosulfate reductase	EC 1.8.4.8	Cytoplasm	SO_3_^2−^ → PAPS
APS kinase	Adenylylsulfate kinase	EC 2.7.1.25	Cytoplasm	PAPS → APS

In addition, sulfite could have pernicious effect on the cell unless it is digested quickly in the organism. So far, the sulfite acceptor oxidoreductase (SAR) that catalyzes sulfite to sulfate has not been identified in the genome of *A. thiooxidans* A01, neither was the *A. thiooxidans* DSM 17318 genome [10]. Phosphoadenosine phosphosulfate (PAPS) reductase and adenylylsulfate (APS) kinase genes were discovered in *A thiooxidans* A01, while the sulfate adenylyltransferase dissimilatory-type (SAT) gene that catalyzed adenosine - 5′ - phosphosulfate (APS) to sulfite was not detected (Figure 6). Therefore, another putative pathway based on PAPS reductase and APS kinase that catalyze sulfite to produce sulfate indirectly is proposed: sulfite is catalyzed by PAPS reductase and APS kinase to generate PAPS and APS in succession, while the latter could produce sulfate as the final product via an unknown mechanism.

Another putative pathway of sulfur oxidation is that the periplasmic sulfite combines with sulfur atom to form thiosulfate spontaneously, without enzymatic catalysis. Thiosulfate:quinone oxidoreductase (TQO) located in the cytomembrane is responsible for the catalysis of thiosulfate to produce tetrathionate. The thiosulfate oxidation catalyzed by TQO was illustrated previously in *A. ambivalens*: DoxD catalyzes thiosulfate to tetrathionate and simultaneously produces two electrons, and then DoxA transfers electrons to the quinone [54]. Tetrathionate hydrolase (TetH), a soluble periplasmic enzyme, is able to catalyze the hydrolysis of tetrathionate [35]. The documented hydrolysates of tetrathionate in *A. thiooxidans* are thiosulfate and sulfate [55]. Furthermore, sulfur atoms (S) may be also one of important hydrolysates, which explains why the expression level of *sor* gene was higher in S^0^ medium.

Thiosulfate could be directly catalyzed by the incomplete Sox complex to generate sulfate [10]. As to the truncated Sox system which is absent of Sox (CD)_2_, the well-studied pathways were provided from some valuable references [9,17,56,57]. Initially, the SoxXA complex oxidatively couples the sulfane sulfur of thiosulfate to a SoxY-cysteine-sulfhydryl group of the SoxYZ complex to form SoxY-thiocysteine-S-sulfate (SoxYZ-S-S-SO_3_^−^). Subsequently, the terminal sulfone (−SO_3_^−^) group is released as sulfate by the activity of the SoxB component to produce S-thiocysteine (SoxYZ-S-S^−^). Due to the lack of the sulfur dehydrogenase Sox (CD)_2_ component, the sulfur atom of the sulfane intermediate (SoxYZ-S-S^−^) is plausibly dropped from SoxYZ or oxidized by the alternative sulfur dioxygenase (SDO) to generate SoxYZ-cysteine-S-sulfate (SoxYZ-S-SO_3_^−^) [9]. Eventually, the sulfonate moiety of SoxYZ-S-SO_3_^−^ is again hydrolyzed by SoxB, regenerating SoxYZ in the process. In addition, other forms of sulfur except for thiosulfate could participate in the Sox pathway via either enzymatic (such as HS^−^) or nonenzymatic (such as S_8_) conjugation to SoxY at the proper intermediate state [17].

There is a strong connection between the expression of the *sox* cluster genes and the terminal oxidase genes. The hypothetical electron pathway is that electrons from the Sox system are transferred via QH_2_ to the terminal oxidases (*bd* and *bo*_*3*_) and the NADH complex (Figure 6).

To date, little is known about electron transfer chains in *A. thiooxidans*, but it has been elaborated in *A. ferrooxidans*. One of the electron transfer chains in *A. ferrooxidans* is that electrons from ferrous iron oxidation flow through Cyc2 to rusticyanin, and then be transferred to oxygen via c-cytochrome Cyc1 to *aa*_3_ cytochrome oxidase (downhill electron pathway) or to NAD^+^ via c-cytochrome CycA1 → *bc*_1_ complex → ubiquinone pool → NADH dehydrogenase (uphill electron pathway) [2,6,58]. Another is that electrons from elemental sulfur or RISCs are transferred via the quinol pool (QH_2_) either (1) directly to terminal oxidases *bd* or *bo*_3_, or indirectly to a *bc*_1_ complex and a cytochrome c (CycA2) or a high potential iron-sulfur protein (HiPIP), whose gene *iro* was identified to be associated with the pet II gene cluster thought to be involved in sulfur oxidation, probably to the *aa*_3_ oxidase to produce a proton gradient or (2) to NADH complex I to generate reducing power [2,6]. However, *A. thiooxidans* has only the sulfur oxidation system and annotated results showed that *bo*_3_-type terminal oxidases and *bd*-type terminal oxidases exist in the draft genome of *A. thiooxidans* A01. Thus, the hypothesized electron transfer chain in *A. thiooxidans* A01 is proposed: electrons from SQR, TQO, Sox compounds and HDR are transferred through the QH_2_ either to terminal oxidase (*bd* and *bo*_3_) to produce proton gradient, or to NADH complex I to generate reducing power.

## Conclusion

Bioinformatics analysis of the genome sequence of *A. thiooxidans* A01 provides a valuable platform for gene discovery and functional prediction that is much important given the difficulties in performing standard genetic research in this microorganism. Based on our analysis and available documented data, a hypothetical model for sulfur oxidation and electron transportation is proposed with several distinguished features. The elemental sulfur (S_8_) in the outer membrane is activated and transported into the periplasm as thiol-bound sulfane sulfur atoms (R-S-S_n_H). And then, the R-S-S_n_H is further oxidized in the periplasm where SDO, TetH, and Sox system perform their functions. The cytoplasmic membrane involving SQR and TQO is the third region with electrons transferring. In the cytoplasm, the sulfur-containing metabolites are catalyzed to eventually produce sulfate by a series of enzymes. Therefore, this study provides novel insights and more instructive guides into sulfur oxidation metabolism in *A. thiooxidans*. However, many fundamental questions remain unanswered. For example, some genes involved in the sulfur oxidation, such as *sor* gene, need to be further verified via biochemical experiments, and it is critical to determine the features of key metabolic enzymes involved in sulfur oxidation mechanism, which warrants further investigations of this organism in the future.

## Competing interests

The authors declare that they have no competing interests.

## Authors’ contributions

HY, XZ, XL, and YL designed the experiments, XZ and XL performed the experiments, HY, XZ and ZH analyzed the data, XG and QH carried out the annotation of draft genome sequence, YX, JC, LM and JN participated in molecular biologic experiments, XZ wrote the manuscript, XL, ZH and HY revised the manuscript. All authors read and approved the final manuscript.

## References

[B1] Hallberg KB González-Toril E Johnson DB *Acidithiobacillus ferrivorans*, sp. nov.; facultatively anaerobic, psychrotolerant iron-, and sulfur-oxidizing acidophiles isolated from metal mine-impacted environments Extremophiles 2010 14 1 9 19 19787416 10.1007/s00792-009-0282-y

[B2] Quatrini R Appia-Ayme C Denis Y Jedlicki E Holmes DS Bonnefoy V Extending the models for iron and sulfur oxidation in the extreme acidophile *Acidithiobacillus ferrooxidans* BMC Genomics 2009 10 394 19703284 10.1186/1471-2164-10-394 PMC2754497

[B3] Rohwerder T Gehrke T Kinzler K Sand W Bioleaching review part A: progress in bioleaching: fundamentals and mechanisms of bacterial metal sulfide oxidation Appl Microbiol Biotechnol 2003 63 239 248 14566432 10.1007/s00253-003-1448-7

[B4] Holmes DS Bonnefoy V Rawlings DE, Johnson DB Genetic And Bioinformatic Insights Into Iron And Sulfur Oxidation Mechanisms Of Bioleaching Organisms Biomining 2007 Berlin: Springer 281 307

[B5] Pronk JT Meulenberg R Hazeu W Bos P Kuenen JG Oxidation of reduced inorganic sulphur compounds by acidophilic thiobacilli FEMS Microbiol Lett 1990 75 2–3 293 306

[B6] Valdes J Pedroso I Quatrini R Dodson RJ Tettelin H Blake R Eisen JA Holmes DS *Acidithiobacillus ferrooxidans* metabolism: from genome sequence to industrial applications BMC Genomics 2008 9 597 19077236 10.1186/1471-2164-9-597 PMC2621215

[B7] Steudel R Lens PNL, Pol LH The Chemical Sulfur Cycle Environmental Technologies to Treat Sulfur Pollution 2000 IWA Publishing: London 1 31

[B8] Rohwerder T Sand W The sulfane sulfur of persulfides is the actual substrate of the sulfur-oxidizing enzymes from *Acidithiobacillus* and *Acidiphilium* spp Microbiology 2003 149 7 1699 1710 12855721 10.1099/mic.0.26212-0

[B9] Chen L Ren Y Lin J Liu X Pang X Lin J *Acidithiobacillus caldus* sulfur oxidation model based on transcriptome analysis between the wild type and sulfur oxygenase reductase defective mutant PLoS One 2012 7 9 e39470 22984393 10.1371/journal.pone.0039470 PMC3440390

[B10] Bobadilla Fazzini RA Cortés MP Padilla L Maturana D Budinich M Maass A Parada P Stoichiometric modeling of oxidation of reduced inorganic sulfur compounds (Riscs) in *Acidithiobacillus thiooxidans* Biotechnol Bioeng 2013 110 8 2242 2251 23436458 10.1002/bit.24875

[B11] Mangold S Valdes J Holmes DS Dopson M Sulfur metabolism in the extreme acidophile *Acidithiobacillus caldus* Front Microbiol 2011 2 17 21687411 10.3389/fmicb.2011.00017 PMC3109338

[B12] Schütz M Maldener I Griesbeck C Hauska G Sulfide-quinone reductase from *Rhodobacter capsulatus*: requirement for growth, periplasmic localization, and extension of gene sequence analysis J Bacteriol 1999 181 6516 6523 10515944 10.1128/jb.181.20.6516-6523.1999 PMC103789

[B13] Friedrich CG Poole RK Physiology And Genetics Of Bacterial Sulfur Oxidation Advances in Microbial Physiology 1997 Amsterdam: Elsevier 235 289

[B14] Friedrich CG Rother D Bardischewsky F Quentmeier A Fischer J Oxidation of reduced inorganic sulfur compounds by bacteria: emergence of a common mechanism? Appl Environ Microbiol 2001 67 7 2873 2882 11425697 10.1128/AEM.67.7.2873-2882.2001 PMC92956

[B15] Friedrich CG Quentmeier A Bardischewsky F Rother D Kraft R Kostka S Prinz H Novel genes coding for lithotrophic sulfur oxidation of *Paracoccus pantotrophus* GB17 J Bacteriol 2000 182 17 4677 4687 10940005 10.1128/jb.182.17.4677-4687.2000 PMC111341

[B16] Rother D Henrich H Quentmeier A Bardischewsky F Friedrich CG Novel genes of the *sox* gene cluster, mutagenesis of the flavoprotein SoxF, and evidence for a general sulfur-oxidizing system in *Paracoccus pantotrophus* GB17 J Bacteriol 2001 183 15 4499 4508 11443084 10.1128/JB.183.15.4499-4508.2001 PMC95344

[B17] Ghosh W Dam B Biochemistry and molecular biology of lithotrophic sulfur oxidation by taxonomically and ecologically diverse bacteria and archaea FEMS Microbiol Rev 2009 33 6 999 1043 19645821 10.1111/j.1574-6976.2009.00187.x

[B18] de Jong GAH Hazeu W Bos P Kuenen JG Polythionate degradation by tetrathionate hydrolase of *Thiobacillus ferrooxidans* Microbiology 1997 143 part 2 499 504 10.1099/00221287-143-2-499 33711857

[B19] Rzhepishevska OI Valdes J Marcinkeviciene L Gallardo CA Meskys R Bonnefoy V Holmes DS Dopson M Regulation of a novel *Acidithiobacillus caldus* gene cluster involved in metabolism of reduced inorganic sulfur compounds Appl Environ Microbiol 2007 73 22 7367 7372 17873067 10.1128/AEM.01497-07 PMC2168230

[B20] Gardner MN Rawlings DE Production of rhodanese by bacteria present in bio-oxidation plants used to recover gold from arsenopyrite concentrates J Appl Microbiol 2000 89 1 185 190 10945796 10.1046/j.1365-2672.2000.01117.x

[B21] Yoch DC Lindstrom ES Survey of the photosynthetic bacteria for rhodanese (thiosulfate: cyanide sulfur transferase) activity J Bacteriol 1971 106 2 700 701 5573738 10.1128/jb.106.2.700-701.1971 PMC285151

[B22] Valdés J Pedroso I Quatrini R Holmes DS Comparative genome analysis of *Acidithiobacillus ferrooxidans.****A. thiooxidans *****and *****A. caldus*****: Insights into their metabolism and ecophysiology** Hydrometallurgy 2008 94 1–4 180 184

[B23] Quatrini R Valdès J Jedlicki E Holmes DS Quatrini R, Valdès J, Jedlicki E, Holmes DS The Use Of Bioinformatics And Genome Biology To Advance Our Understanding Of Bioleaching Microorganisms Microbial Processing of Metal Sulfides 2007 Berlin: Springer 221 239

[B24] Huang X Zhao M Liu W Guan Y Shi Y Wang Q Wu S He M Gigabase-scale transcriptome analysis on four species of pearl oysters Mar Biotechnol 2013 15 3 253 264 23011005 10.1007/s10126-012-9484-x

[B25] Delcher AL Harmon D Kasif S White O Salzberg SL Improved microbial gene identification with GLIMMER Nucleic Acids Res 1999 27 23 4636 4641 10556321 10.1093/nar/27.23.4636 PMC148753

[B26] Bedell JA Korf I Gish W MaskerAid: a performance enhancement to RepeatMasker Bioinformatics 2000 16 11 1040 1041 11159316 10.1093/bioinformatics/16.11.1040

[B27] Lagesen K Hallin P Rødland EA Stærfeldt H Rognes T Ussery DW RNAmmer: consistent and rapid annotation of ribosomal RNA genes Nucleic Acids Res 2007 35 9 3100 3108 17452365 10.1093/nar/gkm160 PMC1888812

[B28] Lowe TM Eddy SR tRNAscan-SE: a program for improved detection of transfer RNA genes in genomic sequence Nucleic Acids Res 2003 25 5 955 964 9023104 10.1093/nar/25.5.955 PMC146525

[B29] Silverman MP Lundgren DG Studies on the chemoautotrophic iron bacterium *Ferrobacillus ferrooxidans*: An improved medium and a harvesting procedure for securing high cell yields J Bacteriol 1959 77 5 642 647 13654231 10.1128/jb.77.5.642-647.1959 PMC290434

[B30] Ramírez P Guiliani N Valenzuela L Beard S Jerez CA Differential protein expression during growth of *Acidithiobacillus ferrooxidans* on ferrous iron, sulfur compounds, or metal sulfides Appl Environ Microbiol 2004 70 8 4491 4498 15294777 10.1128/AEM.70.8.4491-4498.2004 PMC492426

[B31] Simms D Cizdziel P Chomczynski P TRIzol^TM^: A new reagent for optimal single-step isolation of RNA Focus 1993 15 4 99 102

[B32] Livak KJ Schmittgen TD Analysis of relative gene expression data using real-time quantitative PCR and the 2^−ΔΔCT^ method Methods 2001 25 4 402 408 11846609 10.1006/meth.2001.1262

[B33] Suzuki I Oxidation of inorganic sulfur compounds: chemical and enzymatic reactions Can J Microbiol 1999 45 2 97 105

[B34] Zhang L Liu X Liu J Zhang Z Characteristics and function of sulfur dioxygenase in echiuran worm *Urechis unicinctus* PLoS One 2013 8 12 e81885 24312599 10.1371/journal.pone.0081885 PMC3846777

[B35] Bugaytsova Z Lindström EB Localization, purification and properties of a tetrathionate hydrolase from *Acidithiobacillus caldus* Eur J Biochem 2004 271 2 272 280 14717695 10.1046/j.1432-1033.2003.03926.x

[B36] Wodara C Kostka S Egert M Kelly DP Friedrich CG Identification and sequence analysis of the *soxB* gene essential for sulfur oxidation of *Paracoccus denitrificans* GB17 J Bacteriol 1994 176 20 6188 6191 7928987 10.1128/jb.176.20.6188-6191.1994 PMC196957

[B37] Beller HR Chain PSG Letain TE Chakicherla A Larimer FW Richardson PM Coleman MA Wood AP Kelly DP The genome sequence of the obligately chemolithoautotrophic, facultatively anaerobic bacterium *Thiobacillus denitrificans* J Bacteriol 2006 188 4 1473 1488 16452431 10.1128/JB.188.4.1473-1488.2006 PMC1367237

[B38] Kletzin A Molecular characterization of the *sor* gene, which encodes the sulfur oxygenase/reductase of the thermoacidophilic Archaeum *Desulfurolobus ambivalens* J Bacteriol 1992 174 18 5854 5859 1522063 10.1128/jb.174.18.5854-5859.1992 PMC207119

[B39] He Z Li Y Zhou P Liu S Cloning and heterologous expression of a sulfur oxygenase/reductase gene from the thermoacidophilic archaeon *Acidianus* sp. S5 in *Escherichia coli* FEMS Microbiol Lett 2000 193 2 217 221 11111027 10.1111/j.1574-6968.2000.tb09427.x

[B40] Pelletier N Leroy G Guiral M Giudici-Orticoni M Aubert C First characterisation of the active oligomer form of sulfur oxygenase reductase from the bacterium *Aquifex aeolicus* Extremophiles 2008 12 2 205 215 18060346 10.1007/s00792-007-0119-5

[B41] Chen ZW Liu YY Wu JF She Q Jiang CY Liu SJ Novel bacterial sulfur oxygenase reductases from bioreactors treating gold-bearing concentrates Appl Microbiol Biotechnol 2007 74 3 688 698 17111141 10.1007/s00253-006-0691-0

[B42] Kletzin A Coupled enzymatic production of sulfite, thiosulfate, and hydrogen sulfide from sulfur: purification and properties of a sulfur oxygenase reductase from the facultatively anaerobic archaebacterium *Desulfurolobus ambivalens* J Bacteriol 1989 171 3 1638 1643 2493451 10.1128/jb.171.3.1638-1643.1989 PMC209792

[B43] Kletzin A Urich T Müller F Bandeiras TM Gomes CM Dissimilatory oxidation and reduction of elemental sulfur in thermophilic archaea J Bioenerg Biomembr 2004 36 1 77 91 15168612 10.1023/b:jobb.0000019600.36757.8c

[B44] Emmel T Sand W König WA Bock E Evidence for the existence of a sulphur oxygenase in *Sulfolobus brierleyi* Microbiology 1986 132 12 3415 3420

[B45] Li M Chen Z Zhang P Pan X Jiang C An X Liu S Chang W Crystal structure studies on sulfur oxygenase reductase from *Acidianus tengchongensis* Biochem Biophys Res Commun 2008 369 3 919 923 18329378 10.1016/j.bbrc.2008.02.131

[B46] Urich T Bandeiras TM LEAL SS Rachel R Albrecht T Zimmermann P Scholz C Teixeira M Gomes CM Kletzin A The sulphur oxygenase reductase from *Acidianus ambivalens* is a multimeric protein containing a low-potential mononuclear non-haem iron centre Biochem J 2004 381 137 146 15030315 10.1042/BJ20040003 PMC1133771

[B47] Urich T Kroke A Bauer C Seyfarth K Reuff M Kletzin A Identification of core active site residues of the sulfur oxygenase reductase from *Acidianus ambivalens* by site-directed mutagenesis FEMS Microbiol Lett 2005 248 2 171 176 15970399 10.1016/j.femsle.2005.05.031

[B48] Valdes J Quatrini R Hallberg K Dopson M Valenzuela PDT Holmes DS Draft genome sequence of the extremely acidophilic bacterium *Acidithiobacillus caldus* ATCC 51756 reveals metabolic versatility in the genus *Acidithiobacillus* J Bacteriol 2009 191 18 5877 5878 19617360 10.1128/JB.00843-09 PMC2737959

[B49] Valdes J Ossandon F Quatrini R Dopson M Holmes DS Draft genome sequence of the extremely acidophilic biomining bacterium *Acidithiobacillus thiooxidans* ATCC 19377 provides insights into the evolution of the *Acidithiobacillus* genus J Bacteriol 2011 193 24 7003 7004 22123759 10.1128/JB.06281-11 PMC3232857

[B50] Chen Z Jiang C She Q Liu S Zhou P Key role of cysteine residues in catalysis and subcellular localization of sulfur oxygenase-reductase of *Acidianus tengchongensis* Appl Environ Microbiol 2005 71 2 621 628 15691910 10.1128/AEM.71.2.621-628.2005 PMC546804

[B51] Urich T Gomes CM Kletzin A Frazão C X-ray structure of a self-compartmentalizing sulfur cycle metalloenzyme Science 2006 311 5763 992 996 16484493 10.1126/science.1120306

[B52] Urich T Coelho R Kletzina A Frazao C The sulfur oxygenase reductase from *Acidianus ambivalens* is an icosatetramer as shown by crystallization and Patterson analysis BBA-Proteins Proteomics 2005 1747 2 267 270 15698962 10.1016/j.bbapap.2004.11.015

[B53] Schilling CH Palsson BO Assessment of the metabolic capabilities of *Haemophilus influenzae* Rd through a genome-scale pathway analysis J Theor Biol 2000 203 3 249 283 10716908 10.1006/jtbi.2000.1088

[B54] Müller FH Bandeiras TM Urich T Teixeira M Gomes CM Kletzin A Coupling of the pathway of sulphur oxidation to dioxygen reduction: characterization of a novel membrane-bound thiosulphate:quinone oxidoreductase Mol Microbiol 2004 53 4 1147 1160 15306018 10.1111/j.1365-2958.2004.04193.x

[B55] Tano T Kitaguchi H Harada M Nagasawa T Sugio T Purification and some properties of a tetrathionate decomposing enzyme from *Thiobacillus thiooxidans* Biosci Biotechnol Biochem 1996 60 224 227 10.1271/bbb.60.224 27299398

[B56] Meyer B Imhoff JF Kuever J Molecular analysis of the distribution and phylogeny of the *soxB* gene among sulfur-oxidizing bacteria – evolution of the Sox sulfur oxidation enzyme system Environ Microbiol 2007 9 12 2957 2977 17991026 10.1111/j.1462-2920.2007.01407.x

[B57] Ghosh W Mallick S DasGupta SK Origin of the Sox multienzyme complex system in ancient thermophilic bacteria and coevolution of its constituent proteins Res Microbiol 2009 160 6 409 420 19616092 10.1016/j.resmic.2009.07.003

[B58] Brasseur G Levican G Bonnefoy V Holmes D Jedlicki E Lemesle-Meunier D Apparent redundancy of electron transfer pathways via *bc*_*1*_ complexes and terminal oxidases in the extremophilic chemolithoautotrophic *Acidithiobacillus ferrooxidans* Biochim Biophys Acta-Bioenerg 2004 1656 2–3 114 126 10.1016/j.bbabio.2004.02.008 15178473

